# High-temperature continuous-wave laser realized in hollow microcavities

**DOI:** 10.1038/srep07180

**Published:** 2014-11-24

**Authors:** Zhifeng Shi, Yuantao Zhang, Xijun Cui, Shiwei Zhuang, Bin Wu, Xin Dong, Baolin Zhang, Guotong Du

**Affiliations:** 1 State Key Laboratory on Integrated Optoelectronics, College of Electronic Science and Engineering, Jilin University, Qianjin Street 2699, Changchun 130012, China

## Abstract

Recently, an urgent requirement of ultraviolet (UV) semiconductor laser with lower cost and higher performance has motivated our intensive research in zinc oxide (ZnO) material owing to its wide direct band gap and large exciton binding energy. Here, we demonstrate for the first time continuous-wave laser in electrically-pumped hollow polygonal microcavities based on epitaxial ZnO/MgO-core/shell nanowall networks structures, and whispering gallery type resonant modes are responsible for the lasing action. The laser diodes exhibit an ultralow threshold current density (0.27 A/cm^2^), two or three orders of magnitude smaller than other reported UV-light semiconductor laser diodes to our knowledge. More importantly, the continuous-current-driven diode can achieve lasing up to ~430 K, showing a good temperature tolerance. This study indicates that nano-size injection lasers can be made from epitaxial semiconductor microcavities, which is a considerable advance towards the realization of practical UV coherent light sources, facilitating the existing applications and suggesting new potentials.

Short-wavelength semiconductor lasers are considered as the next-generation laser sources because of their promise in high-density data-storage, communications, integrated circuit miniaturization, medical diagnostics and therapy, and other applications^1^,^2^,^3^,^4^. To date, most reported lasers working in the ultraviolet (UV) region are realized by optical pumping^4^,^5^,^6^,^7^, or electrical pumping with a large threshold and a pulsed current mode^8^,^9^, which seriously defer their practical applications and commercialization. To be employed more widely in technologies ranging from information to optoelectronic fields, researches of electrically pumped lasers in a continuous-wave (CW) mode, are highly desired and necessary to be promoted. Besides, of particular concern are their low-threshold characteristic and high stability working in harsh environments, which are important pursuits and certainly worthwhile subjects. Because of its large direct band gap (3.37 eV) and high exciton binding energy (60 meV), ZnO has recently been regarded as one of the most promising material candidates for UV laser diodes^10^,^11^,^12^,^13^,^14^. Also, its exciton stability opens opportunities for making low-threshold excitonic lasing operable at room-temperature (RT) and even higher. A potentially ideal building block for these lasers is highly uniform core/shell (CS) configurations based on low-dimensional ZnO nanostructures. Some features of the CS structure provide more a chance for an enhanced carrier recombination efficiency^15^, such as the effective surface passivation, increased junction area and enhanced carrier confinement, additionally, it greatly stabilizes the device operation at elevated temperatures. Such CS configurations have been intensively studied by many researchers based on low-dimensional nanostructures. For instance, Qian *et al.*^16^ fabricated multi-quantum-well CS nanowire heterostructures based on well-defined III-nitride materials that enabled lasing over a broad range of wavelengths at RT. Hua *et al.*^17^ reported a near-infrared lasing in highly uniform GaAs/GaAsP coaxial CS nanowires, operating at a temperature up to 125 K with a lowered threshold of 8.4 kW/cm^2^. Chen *et al.*^4^ investigated the emission from localized excitons inside a ZnO CS structure, revealing the structure to be stable and insensitive to temperature compared with as-grown ZnO nanowires. And further they observed coherent random lasing from such CS nanowires with a pumping threshold of 0.4 MW/cm^2^. Very recently, Grivas *et al.*^18^ demonstrated a single-mode tunable laser emission from colloidal CdSe/CdS-CS quantum rods deposited on silica microspheres. The specific CS architecture and band structure strongly localized holes in the CdSe core, and a two-dimensional (2-D) quantum confinement of electrons across the elongated shell, favoring a low threshold and a single-mode laser emission.

As a novel nanostructure, the research on 2-D ZnO nanowall networks (NNWs) based CS structure is rare. If the typical 2-D ZnO NNWs-based CS structures can be used to fabricate the optoelectronic devices, high performance nanoscale laser diodes could be developed. This is mainly because of the distinct morphology of 2-D NNWs, in which a series of hollow polygonal microcavities are built by the vertically interconnected nanowalls. Such self-formed closed-loop microcavities favor a whispering gallery type resonant mode owing to the feedback around the interface between ZnO and the air surrounding, which can greatly enhance the oscillator strength of confined light^19^,^20^, while still allowing for optical gain and amplified spontaneous emission at high-temperature (HT). Besides, the NNWs can also easily and rapidly release heat from the device units because of their relatively large surface-volume ratio. Thus a low-threshold excitonic lasing with improved temperature stability can be expected. In the present study, we demonstrate the synthesis, morphology and structural properties of ZnO/MgO-CS NNWs, and construct a metal/oxide/semiconductor-type laser diode. The studied diode exhibits an ultralow threshold current density of 0.27 A/cm^2^, two or three orders of magnitude smaller than other reported UV-light semiconductor laser diodes to our knowledge^8^,^9^,^11^,^13^. More importantly, the coherent continuous-current-driven laser can be sustained at a temperature up to ~430 K. We tentatively discuss the detailed reasons for the low-threshold lasing operating at HT.

## Results

### Characterization analysis of ZnO NNWs

**Figure 1:**
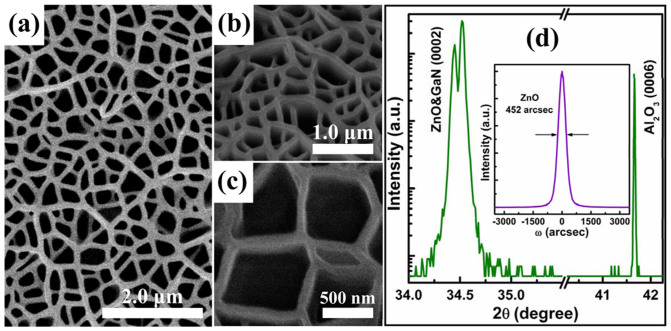
Characterization analysis of ZnO NNWs. (a) Top-view SEM image of well-aligned ZnO NNWs. (b, c) 25°-tilted SEM images at different magnifications. (d) XRD 2*θ* scans of ZnO NNWs. The inset shows the (0002) *ω*-rocking curves of ZnO (0002) reflection.

**Figure 2:**
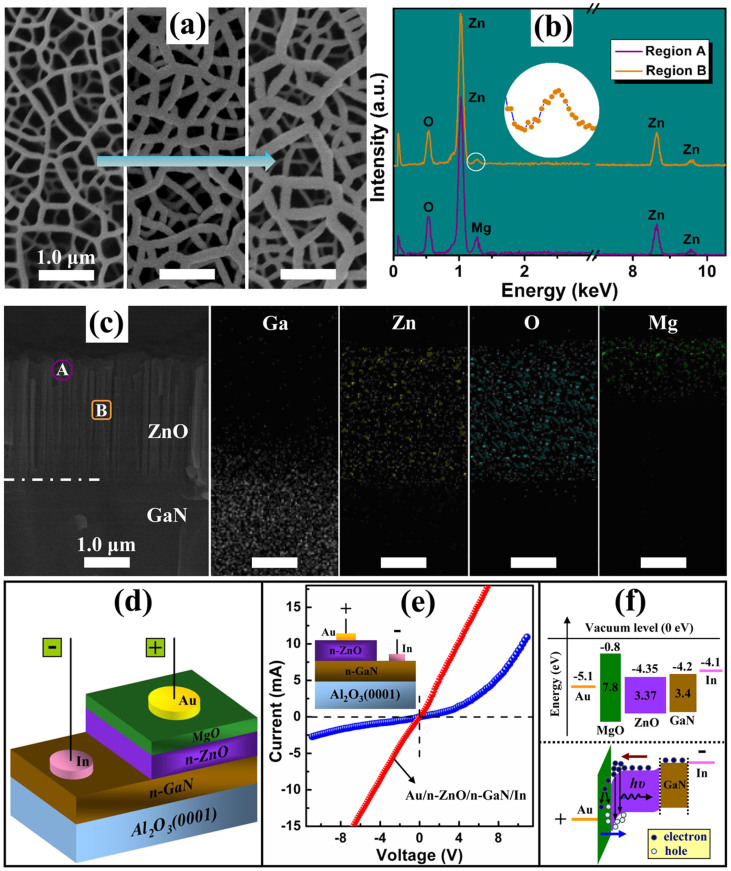
Evidences for ZnO/MgO-CS NNWs structure and band alignment of the heterostructure. (a) SEM images of ZnO NNWs, and ZnO NNWs coated successively with MgO and Au layers (from left to right). (b) EDS spectra obtained from regions A and B marked in (c). (c) Cross-sectional SEM image of MgO-coated ZnO NNWs and corresponding elemental mapping images. The regions marked as “A” and “B” correspond to the head and body of the nanowalls, respectively. (d) Schematic diagram of the studied laser diode. (e) *I*–*V* characteristics of the laser diode (blue line) and the n-ZnO NNWs/n-GaN structure (red line). (f) Simplified band alignment of the Au/MgO/ZnO/GaN/In structure (upper pane), showing the electrical parameters of the involved materials, and the schematic energy band under forward bias (lower pane).

Fig. 1a–c show the typical scanning electron microscope (SEM) images of the produced ZnO NNWs. The nanowalls interconnect with each other and form an uniform network over the whole surface area of the substrate, resembling the typical features observed by Ng *et al*^21^. The 25°-tilted-view SEM image (Fig. 1b) shows that the nanowalls are vertically aligned and have smooth sidewall profiles. More importantly, a series of micro-holes without uniform shape distribution are formed among the interconnected nanowalls. A high-magnification SEM image (Fig. 1c) reveals that the adjacent walls form angles at their junctions, developing polygons with different domain sizes. The resulting micro-holes are straight and extend almost through the whole ZnO layer to the supporting template, which can be clearly observed in the cross-sectional SEM image shown in Fig. 2c. In addition, X-ray diffraction (XRD) measurements were performed to examine the structural properties of the ZnO NNWs. As shown in Fig. 1d, the wide-range XRD 2*θ* patterns are dominated by the diffraction peaks from ZnO (0002) and GaN (0002) planes; no other peaks are detected except for the phases from the Al_2_O_3_ substrate. Unlike most other reports^19^,^22^, the ZnO NNWs obtained here are well-aligned and behave as a pure hexagonal wurtzite phase with a highly preferred *c*-axis orientation. To check the degree of alignment of ZnO NNWs to the normal of substrate, we also performed (0002) *ω*-rocking curve measurements (inset of Fig. 1d), revealing a small full width at half-maximum (FWHM) of 0.1256° (452 arcsecond). This result indicates an excellent ordering of the well-aligned NNWs along the growth direction.

### Identification of the ZnO/MgO-CS NNWs structure

To prepare the metal/oxide/semiconductor structure, we successively deposited insulating MgO (40 nm) and Au (30 nm) layers on the ZnO NNWs. To monitor the epitaxial process, we performed the SEM measurements and the corresponding results are shown in Fig. 2a. Combined with the SEM images and the plane scanning energy-dispersive X-ray spectroscopy (EDS) results (Supplementary Fig. S1) in corresponding preparation steps, we observe that the MgO and Au layers do not fully fill up the micro-holes; they only cover the head of the walls. A regular variation in wall thickness in the lateral direction after each layer stack indicates the formation of a CS geometric configuration. We further investigate this structural feature by using the spot scanning EDS measurements (seen in Fig. 2b) on the head and body of vertical nanowalls, marked as “A” and “B” in Fig. 2c. Here, we take the MgO-coated ZnO NNWs as the research object. The Mg signal in region B has a much lower intensity than that obtained in region A, indicating the formation of ZnO/MgO-CS NNWs structures and a decreasing MgO thickness along the *c*-axis direction. Additional analysis using the EDS mapping conducted on the cross-sectional MgO-coated ZnO NNWs identifies clearly the spatial distributions of Ga, Zn, O and Mg elements exclusively. The resulting CS structure makes ZnO NNWs an ideal template for assembling 2-D functional nanoscale networks. Such morphology characteristics are significantly different from previously reported CS structures that employed 1-D nanostructures as the building blocks, in which all produced CS units are well-separated^4^,^17^. In our case, the 2-D NNWs-based CS structure can be regarded as an integrated unit because of its interconnection behavior. The effective coating of MgO around the ZnO active layer is believed to be favorable for passivating the surface defects of ZnO, blocking the surface-mediated nonradiative recombination process, and thereby stabilizing the device operation. The photoluminescence (PL) specrta (Supplementary Fig. S2) performed on the ZnO/MgO-CS NNWs with different MgO shell thicknesses verify the enhancement of UV luminescence efficiency by MgO coating.

### *I*–*V* characteristics and band alignment of the studied diode

Fig. 2d presents a schematic illustration of the studied device, where the low-resistance n-GaN is used as the electron transport layer, and ZnO NNWs are employed as the active lasing media. The insulating MgO shell serves as the hole supplying, electron blocking and surface passivation layer simultaneously. Fig. 2e shows the current-voltage (*I*–*V*) characteristics of the constructed NNWs-CS diode, in which the positive voltage is connected to the Au electrode. And a nonlinear rectifying behavior is observed. To rule out the interference from the inherent n-ZnO NNWs/n-GaN structure, as shown in the inset of Fig. 2e, *I*–*V* curve of which was also performed. An absolutely linear curve can be obtained, indicating that the n-GaN layer serves as an electrode and that the Au/MgO/ZnO heterostructure is the only contributor to the nonlinear behavior. Fig. 2f shows a simplified band diagram of the Au/MgO/ZnO/GaN/In structure assuming that there are no imperfections at the hetero-interfaces, and the electrical parameters of the involved materials shown in the upper pane are known from literatures^10^,^23^,^24^,^25^. Under forward bias, the possibility of light emission from the studied Schottky diode can be expected. On the one hand, at a low driving voltage, electrons injected from the n-GaN can be confined at the MgO/ZnO interface because of the large conduction band offset (3.55 eV). On the other hand, a sufficiently high bias enables the generation of additional electrons and holes in the MgO layer because of the high-electric-field-induced impact ionization process considering that almost all the voltage is applied onto the insulating MgO layer^26^. Above a critical driving voltage, the generated holes can be swept into the valence band of ZnO. There, the holes radiatively recombine with electrons accumulated at the MgO/ZnO interface, causing near-band-edge emission and even lasing action.

### Electrically pumped lasing action from the diode

**Figure 3:**
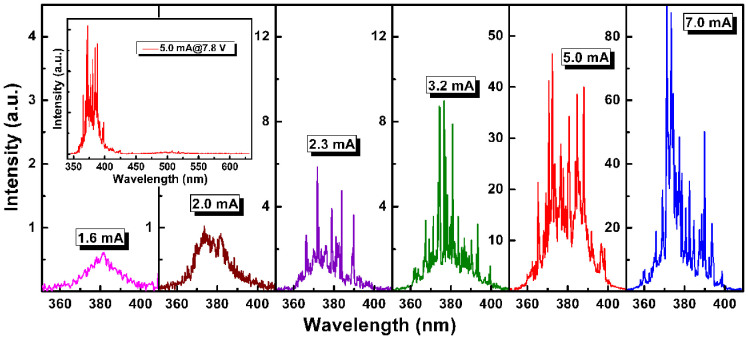
EL spectra of the laser diode obtained under different injection currents. The amplitudes of EL spectra were adjusted for clarity. The inset shows the wide-range EL spectra obtained at 5.0 mA in the UV-visible region.

**Figure 4:**
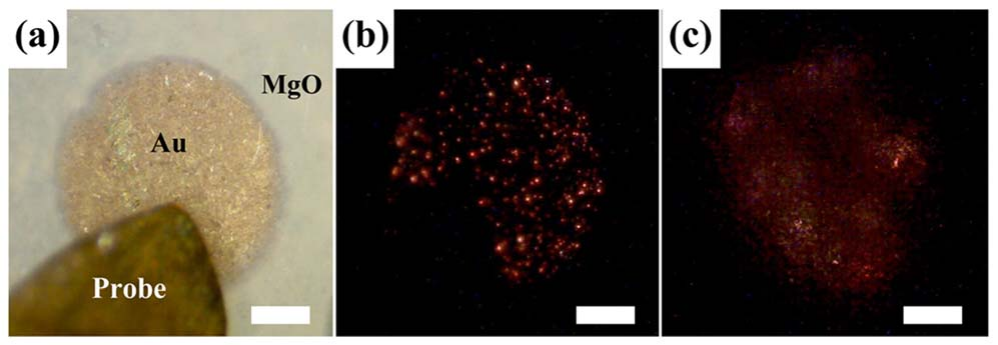
Optical microscopy images of a device unit at 295 K. (a) Microscopy image taken with lamp illumination and zero bias; microscopy images obtained from the (b) front and (c) back of the device unit at 5.8 mA.The scale bar is 250 μm.

For electroluminescence (EL) measurements, the studied NNWs-CS diode is biased at RT (295 K) under continuous-current-injection mode, and a set of surface emission spectra obtained are plotted in Fig. 3. At a low driving current of 1.6 mA, we observe a broad spontaneous emission band centered at ~380 nm with a FWHM of 16.4 nm. Following the increase in current input (2.0 mA), the overall emission intensity increases accordingly. As the current reaches 2.3 mA, some discrete and sharp peaks with FWHMs less than 0.5 nm emerge from the broad spontaneous emission band, implying that the optical gain obtained along the laser cavities is sufficiently high enough to enable lasing^6^,^27^,^28^. With increasing the current to 3.2 mA and above, these discrete and sharp peaks become sharper and more peaks show up. Note that the above phenomena are observed at a steady operation status, and no bias fluctuation action occurs (Supplementary Fig. S3). The threshold current for the proof-of-concept laser diode can be determined from the dependence of the integrated EL intensity *versus* current (Supplementary Fig. S4), and a threshold of ~2.1 mA is derived, corresponding to an ultralow current density of 0.27 A/cm^2^, two or three orders of magnitude smaller than other reported UV-light semiconductor laser diodes, as far as we know^8^,^9^,^11^,^13^. Such a small threshold favors a reduced heating effect and makes it possible to enable high-stability lasing at RT or even higher. Commonly, the mechanism of lasing action realized in ZnO is attributed to excitonic interaction, electron-hole plasma (EHP), or both together^13^. In the present case, excitonic emission ought to bear mostly liability for such lasing action, rather than EHP process, because there is no sign of red-shift for the center of overall lasing envelope with ever-increasing pumping power^29^,^30^. The inset in Fig. 3 shows a wide-range EL spectra obtained at 5.0 mA in the UV-visible region, revealing a negligible defect-related visible emission, the reason of which probably lies in the effective surface passivation by MgO coating. It is generally accepted that the defect emission can impede the UV lasing because the required pumping energy for UV stimulated emission can be consumed with the spontaneous defect emission^1^,^13^. Fig. 4 shows optical microscopy images of the laser diode at RT, and many randomly distributed light spots can be observed from the circular contact area (Fig. 4b), which represent the isolated resonators formed in the ZnO NNWs. Note that the emitted light at ~380 nm is beyond the color range of the digital microscope, inducing color aberration between the captured color (red) and the true emission color (UV). The microscopy image taken from the back of device unit features a quasi-circular halo, likely related to the diffuse reflection effect induced by the single-polished Al_2_O_3_ substrate.

## Discussion

**Figure 5:**
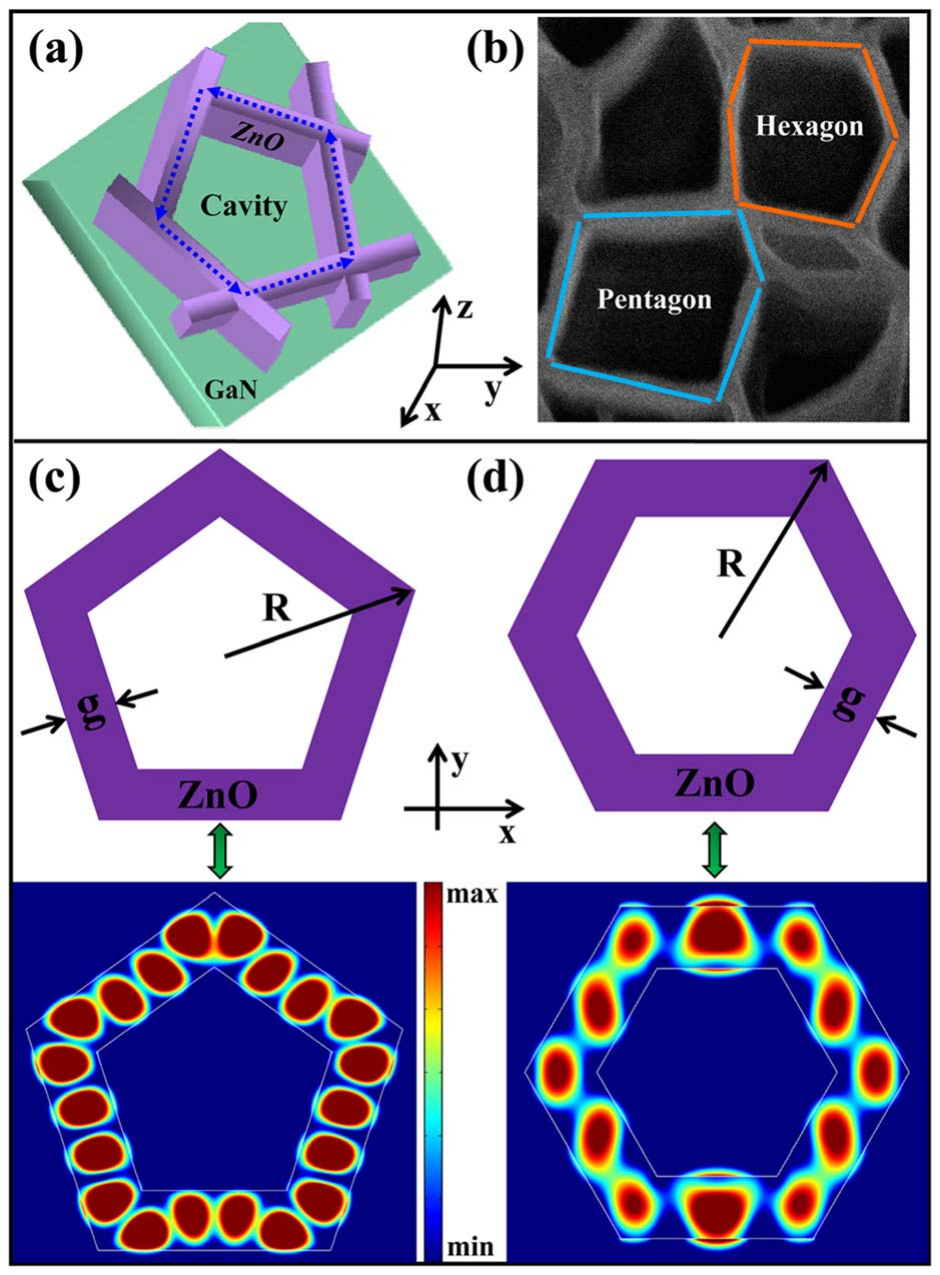
Numerical simulations of Whispering gallery type resonant modes formed in NNWs. (a) Schematic diagram of the self-formed microcavity for optical characterization. (b) Schematic views of the approximate pentagon and hexagon, defined by interconnected nanowalls. (c, d) Simulated resonance patterns diagrams of the proposed pentagon and hexagon nanostructures, displaying that standing wave field distribution can be formed.

An obvious feature of the studied laser diode is the ultralow threshold current density, and it is worthwhile for us to clarify its intrinsic mechanisms because no advanced structures such as double heterojunction or quantum well layers are introduced in this study^11^,^28^. Firstly, the intrinsic characters of ZnO, large exciton binding energy and high optical gain, make a great contribution to the ultralow lasing threshold. Meanwhile, the lasing action through the excitonic interaction process is essentially more easily favorable for a low-threshold laser than that through the EHP process^31^. Secondly, the highly uniform coaxial ZnO/MgO-CS NNWs structures are promising for improving the excitonic emission efficiency, which directly determines the threshold of laser diodes. In virtue of the high dimensionality of ZnO/MgO-CS NNWs structure and effective surface passivation effect, the surface-mediated nonradiative recombination and deep-level emission (DLE) are substantially suppressed. Besides, some other features of the ZnO/MgO-CS NNWs structures provide more a chance for an enhanced carrier recombination efficiency, including an increased junction area and a strong carrier confinement. Finally, and most importantly, the 2-D ZnO NNWs with a distinctive morphology serve as an active gain medium, where a series of nanowall-formed hollow polygonal cavities greatly enhance the optical gain and induce an amplified spontaneous emission; as the oscillator strength of confined light in the self-formed closed-loop microcavities is sufficiently large, an excitonic lasing action with a low-threshold can be expected. In detail, the microcavities, as vividly depicted in Fig. 5a, can be regarded as having a closed-loop light transmission/oscillation process in the interconnected nanowalls. Because the refractive index of ZnO is larger than that of the MgO shell (1.7) and air (1.0), the photons generated at the ZnO/MgO interface would keep circulating around within the so-called resonators to obtain the optical gain, and the lasing characteristics of the present structure are largely based on such a photons oscillation mode. Fig. 5b represents the schematic views of approximate pentagon and hexagon, modeling the hollow closed-loop microcavities above, defined by the interconnected nanowalls. Such polygonal microcavities are remarkably similar to traditional whispering gallery modes in hexagonal micro- and nanorods, in which the total internal reflection of photons at the cavity boundaries supports defined optical oscillation modes^32^,^33^,^34^. 2-D space numerical simulation of whispering gallery type resonant modes, shown in Figs. 5c and d, was also studied using the finite-difference time-domain (FDTD) methods to further demonstrate the lasing characteristics on the basis of photons confinement, oscillation and amplification. The optical fields are well confined near the periphery of the gain medium because of the totally internal reflection at the air/ZnO interface, and distinct standing wave field patterns induced by the feedback at the interfaces are formed. Therefore, we believe that the microcavities formed in the ZnO/MgO-CS NNWs make a great contribution to the ultralow lasing threshold. Nevertheless, it is very difficult for us to define the mode numbers, mode position, and quality factor of the observed lasing behavior because the light transmission/oscillation paths in the proposed closed-loop microcavities are not solely due to their irregular distribution in shape and size; in essence, other circular optical paths or modes may also coexist. Maybe, that is the reason why the detected sharp peaks in the EL spectra under above-threshold currents feature non-uniform spacings between adjacent peaks.

**Figure 6:**
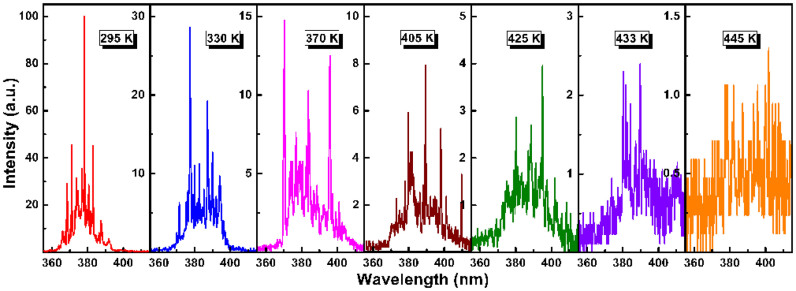
Temperature-dependent EL spectra of the laser diode at a fixed current of 11.5 mA. The amplitudes of EL spectra were adjusted for clarity.

**Figure 7:**
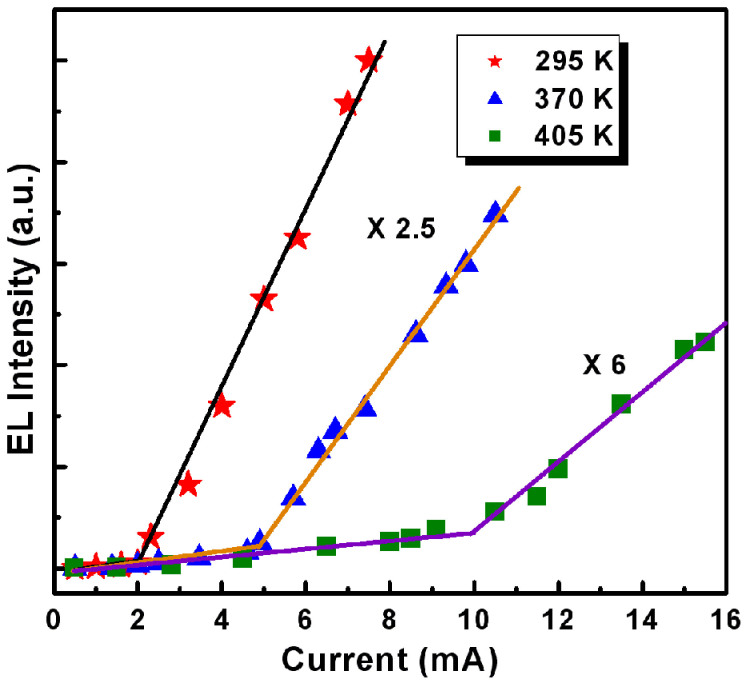
Dependence of the integrated EL intensity on injection current at different operating temperatures. The solid lines are the guides to the eye.

High-quality excitonic lasing does not mean merely a low-threshold feature, and a stable CW operation at RT and even elevated temperatures is also a goal of researchers. Theoretically, the high exciton binding energy of ZnO ensures exciton survival well above RT; and experimentally, optically pumped UV lasing from polycrystalline ZnO has been observed at a temperature up to 550 K^29^,^30^. The above discussions lead to attractive questions: Can the studied laser diode be also operated at HT and what is the upper limit of operating temperature of the studied laser diodes? From an application perspective, if a laser diode that can sustain lasing at HT and be pumped with a CW can be realized, the importance and significance of which will be self-evident. To further verify the sensitivity of the lasing action to temperature, namely stability of the studied diode, temperature-dependent EL measurements were carried out. Over the course of measurement, the working temperature of the entire device chip was adjusted from RT to 445 K, and the driving current was set to 11.5 mA (1.48 A/cm^2^) for comparison. Note that another Au electrode is employed for such measurement. As shown in Fig. 6, the EL performance exhibits a drastic but regular change with increasing the working temperature. As expected, the overall lasing emission band red-shifts with increasing temperature because of the heating-induced band gap shrinkage. Besides, another reason of the transition from excitonic emission to EHP recombination may contribute to the red-shift^30^,^35^,^36^. More importantly, although the emission peaks are observed to diminish and the integrated emission intensity to decrease, the coherent lasing action is efficiently sustained at a high level up to ~430 K, which is also evident from the low FWHM of the sharp modes. Above this critical temperature, the lasing ceases and the light output decreases dramatically, only less than 5% remains. We attribute this reduction of light output to the increasing probability of nonradiative recombination induced by heating effect. At elevated temperatures, a rapid proliferation of structural defects induced by heating effect produces a number of nonradiative recombination centers, reducing the carrier injection efficiency and radiative recombination probability. Such high operating temperature of the studied laser diode is evident of the high quality resonators and rational design of device structure. Up to now, no studies have reported temperature-dependent lasing action from ZnO-based laser diodes, making our results original and useful for progressing towards a practical UV laser with good temperature tolerance. Fig. 7 shows the light-current curves of the laser diode operating at three representative temperature points. The lasing threshold of the diode is well defined by the nonlinearity of the curves. The lasing threshold is found to be 2.1 mA at RT and 4.9 mA at 370 K, further increasing to 10 mA at 405 K. It is reasonable that an absolutely high operating temperature limited for CW lasing should be determined by a weakened gain, and that high pumping power is therefore required to compensate the heating-induced loss.

Although a “softer” threshold behavior is obtained as the studied laser diode is operated at an elevated temperature, an acceptable sensitivity of the threshold to temperature change opens up enormous opportunities for their HT applications. So what are the reasons for the CW lasing action persisting at elevated temperatures? By analyzing of our device structure and experimental results, three possible reasons are summarized and listed as follows: (1) The NNWs are featured with a large surface-volume ratio, implying that the heat could be easily released; and meanwhile, the reabsorption effect of photons, which then again accounts for the heating problem, could be effectively suppressed because the emitted light is more likely to escape from the device. (2) The surface passivation effect of MgO shell layer could improve the excitonic recombination efficiency, which is an exclusive mechanism supporting exciton-related UV lasing and suppressing DLE to overcome the threshold operating at HT. (3) The self-formed closed-loop cavities built by the interconnected nanowalls enhance the oscillator strength of confined light, owing to the whispering gallery type resonant modes, and optical gain could still be acquired at HT. Thus improved temperature stability of the laser diode can be expected.

In conclusion, we have investigated the synthesis, morphology and structural properties of the ZnO/MgO-CS NNWs systematically, and further UV laser diodes based on such structure were fabricated. Because of the naturally self-formed microcavities built by the interconnected nanowalls and surface passivation effects by MgO coating, the laser diode exhibits superior low-threshold characteristics. In addition, we performed the temperature-dependent EL measurements to verify the sensitivity of the lasing action to temperature, and it is found that the studied diode can achieve lasing up to ~430 K. We tentatively discussed the reasons for the stable lasing operating at HT based on the device structure, experimental and simulated results, and a whispering gallery type resonant mode is originally proposed to interpret the lasing characteristics. It is reasonably believed that the ZnO/MgO-CS NNWs structure can serve as good building blocks for fabricating high performance UV laser diodes that can operate well at HT.

## Methods

### Preparation of ZnO/MgO-CS NNWs structures

The coaxial ZnO/MgO-CS NNWs structures were prepared on commercially available n-type GaN/Al_2_O_3_ (0001) substrates, which included the following two steps mainly. Firstly, vertically aligned ZnO NNWs were fabricated by a custom-designed photoassisted metal-organic chemical vapor deposition system without using any catalysts. Diethylzinc (DEZn) and ultrahigh-purity oxygen gas (O_2_) were used as the reactants, with argon as the carrier gas; details on the reaction system can be found elsewhere^37^. In the experiment, a two-step growth method was employed. We precisely controlled the growth parameters, such as the reaction temperature, pressure, and Zn/O ratio to obtain high-quality ZnO NNWs considering that the geometry and morphology of ZnO were very sensitive to growth parameters. In the first step, the growth was conducted at 570 °C for 3 min under a pressure of ~80 Pa, and the flow rates of DEZn and O_2_ were set to 6.5 μmol/min and 8.0 mmol/min, respectively. In the second step, DEZn was typically supplied with a flow rate of 11.4 μmol/min, and the flow rate of O_2_ was remained constant. The main ZnO layer was then grown at 800 °C for 1 hour under a pressure of ~1650 Pa. After the growth process, the susceptor was allowed to cool down to RT and samples removed. Secondly, the samples were transferred into a radio-frequency (rf) magnetron sputtering system (JZCK-IVB) for the MgO shell deposition. Before the deposition, the sputtering chamber was evacuated with a turbo molecular pump to a base pressure below 10^−4^ Pa, then filled with the working gas (argon) to a pressure of 1.0 Pa. A ~40 nm MgO shell layer was deposited by sputtering an MgO ceramic target (99.999%) at the temperature of 500 °C with a rf power of 110 W. During the deposition, the sample holder was rotated (45 rpm) to ensure a uniform shell layer.

### Device preparation

For device preparations, part of the epitaxial layers, consisting of ZnO NNWs and MgO shell layer were removed by dilute hydrochloric acid (~3%) until the n-GaN was exposed for n-type Ohmic contact formation. An In electrode was soldered on the n-GaN side as the cathode contact electrode. Monolayer Au (~30 nm) was thermally evaporated on the MgO shell layer as the anode contact electrode, and patterned into circular pad (1 mm) with a custom shadow mask. Then the device was annealed in nitrogen atmosphere at 380 °C for 3 min to reduce the contact resistance as well as increase contact adhesion by using a quartz tube furnace system (OTF-1200X).

### Characterizations

The microstructures of the products were investigated by SEM (JEOL, JSM-7500F, 15 keV). The crystallinity was determined by XRD (Rigaku Ultima IV) using a Cu *K*α radiation (40 kV, 20 mA). The EDS attached to the SEM was used to study the chemical compositions of the products. PL spectra were recorded with a monochromator/spectrograph (Zolix Omni-λ 500) at RT. The excitation light source was a He-Cd laser with a wavelength of 325 nm and a power of 30 mW. The *I*–*V* characteristics of the devices were measured using a Keithley 2400 semiconductor characterization analyzer and the EL spectra were measured using an acquisition equipment including a photomultiplier tube (PMTH-S1-R1527) and lock-in amplifier systems (Stanford SR830-DSP).

### FDTD simulation

In order to characterize the cavity field distribution and photon localization in the proposed hollow polygonal microcavities, 2-D FDTD was carried out to calculate and simulate the electrical field distribution. The active region was modeled as vertically interconnected NNWs with the wall thickness of 100 nm, and the outer radius of the polygonal configuration was 300 nm. The incident wavelength was defined as λ = 385 nm. The refractive index of ZnO NNWs was set to 2.45.

## Author Contributions

Z.S., Y.Z. and G.D. conceived and designed the experiments. Z.S., X.C. and S.Z. carried out the experiments. B.W. and X.D. performed and analyzed the XRD, SEM and PL measurements. Z.S., Y.Z. and B.Z. conducted the electrically pumped lasing measurement and B.Z. contributed to the data analysis. Z.S., Y.Z. and G.D. co-wrote the paper, and G.D. supervised the project.

## Supplementary Material

Supplementary Information High-temperature continuous-wave laser realized in hollow microcavities
