# Supplementary material for: High-temperature continuous-wave laser realized in hollow microcavities
Source: Sci Rep. 2014 Nov 24;4:7180. doi: 10.1038/srep07180 (PMC4241518; doi:10.1038/srep07180)
Supplement: Supplementary Information — High-temperature continuous-wave laser realized in hollow microcavities [file srep07180-s1.doc]

**High-temperature continuous-wave laser realized in hollow microcavities**

Zhifeng Shi*, Yuantao Zhang*, Xijun Cui, Shiwei Zhuang, Bin Wu, Xin Dong, Baolin Zhang & Guotong Du

^1^State Key Laboratory on Integrated Optoelectronics, College of Electronic Science and Engineering, Jilin University, Qianjin Street 2699, Changchun 130012, China. * These authors contributed equally to this work. Correspondence and requests for materials should be addressed to B.Z. (email: [zbl@jlu.edu.cn](mailto:zbl@jlu.edu.cn)) or G.D. (email: laserlab@jlu.edu.cn).

**Plane scanning EDS results in different preparation steps**


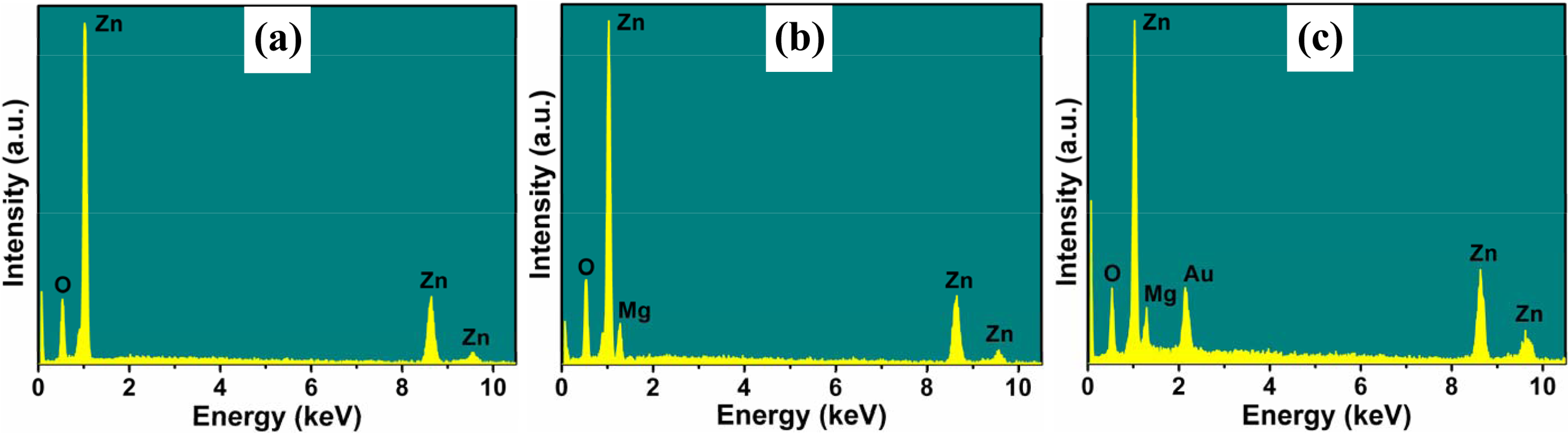


**Figure S1.** Plane scanning EDS results corresponding to different preparation steps: (a) ZnO NNWs; (b, c) ZnO NNWs coated successively with MgO and Au layers.

Fig. S1 presents the plane scanning EDS results of the bare ZnO NNWs, and ZnO NNWs coated successively with MgO and Au layers. The observations of Mg and Au elements in Fig. S1b and c vividly identify the corresponding preparation steps.

**Optical properties of MgO-coated ZnO NNWs**


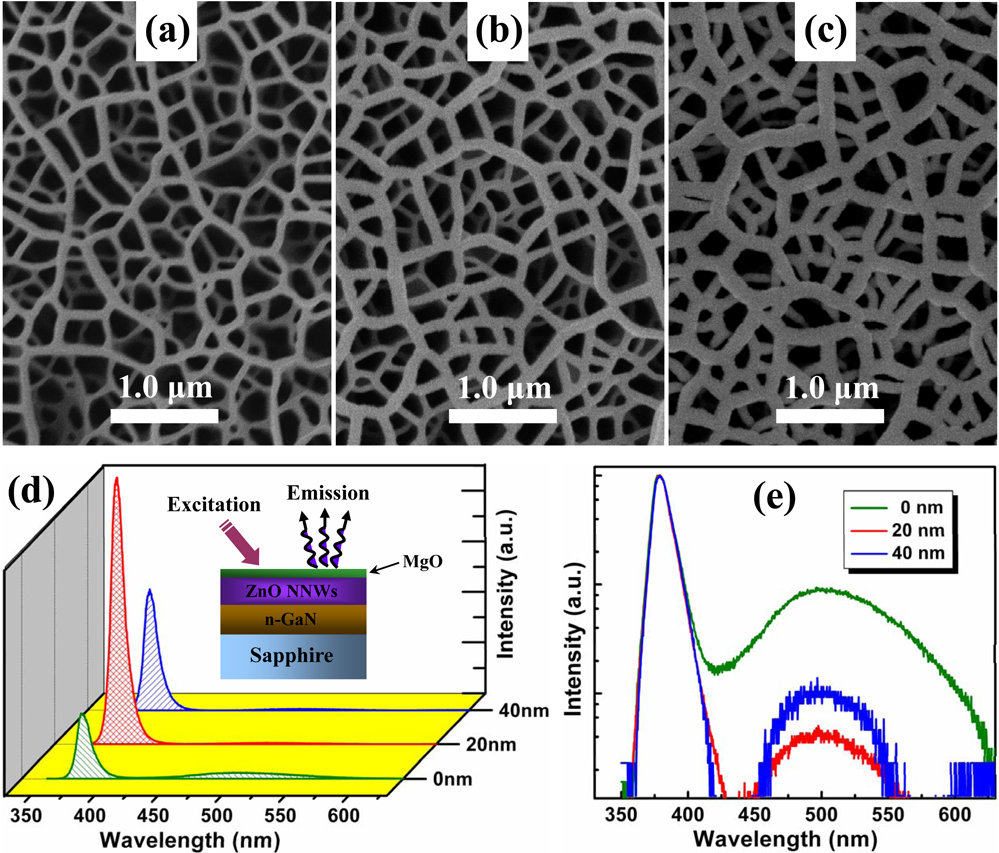


**Figure S2.** SEM images the ZnO/MgO-CS NNWs with different MgO shell thickness: (a) 0 nm, (b) 20 nm, and (c) 40 nm. (d) PL spectra of the three samples performed at RT. (e) Normalized PL spectra of the three samples in a logarithmic scale.

In order to verify the improvement of MgO shell layer on the optical properties of ZnO NNWs, three CS NNWs samples with different MgO shell thickness (0, 20, and 40 nm) were prepared. Fig. S2a-c show the corresponding SEM images of three samples, and a regular variation with a gradually thickened wall thickness is observed. The variation in the optical properties of three samples was investigated by PL measurement and shown in Fig. S2d, and schematic arrangement of the PL measurement configuration is presented in the inset. One can see from the figure that the UV and visible emissions are changed markedly for three samples. The MgO coating enhances the emission intensity of UV component, while passivates that of visible component. For a better understanding, the three PL spectra are normalized in a logarithmic scale shown in Fig. S2e.

**Variation of the bias voltage with time under a fixed driving current of 5.0 mA**


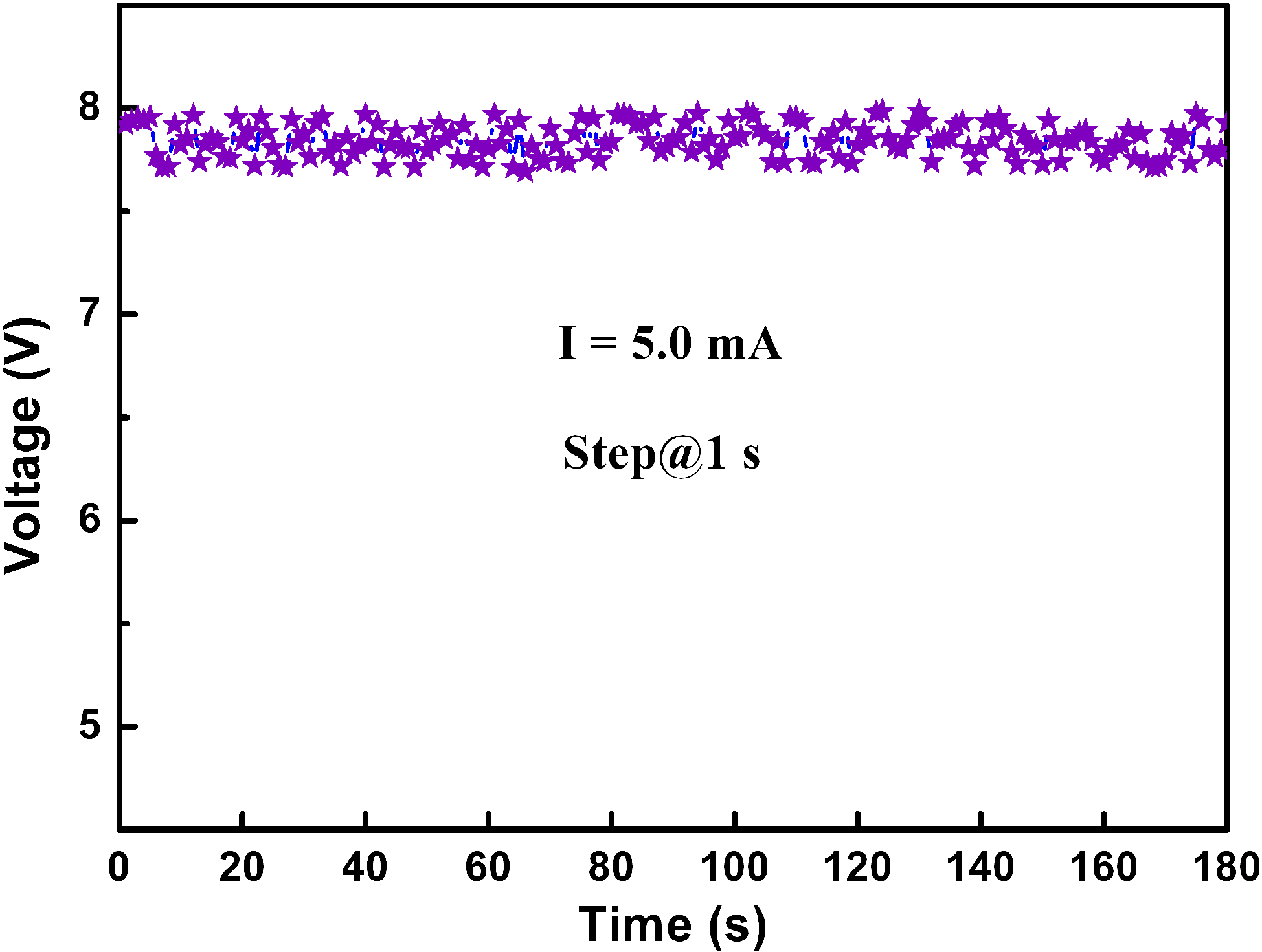


**Figure S3.** Experimental bias voltage of the laser diode as a function of time under a fixed driving current of 5.0 mA.

We performed an experiment on the variation of the bias voltage with time under a fixed driving current of 5.0 mA. As shown in Fig. S3, almost no bias fluctuation can be found during the whole measuring time (3 min), which is significantly longer than the single EL testing time (about 50 s), indicating a steady bias when the studied diode is operated.

**Dependence of the integrated EL intensity on injection current at RT**


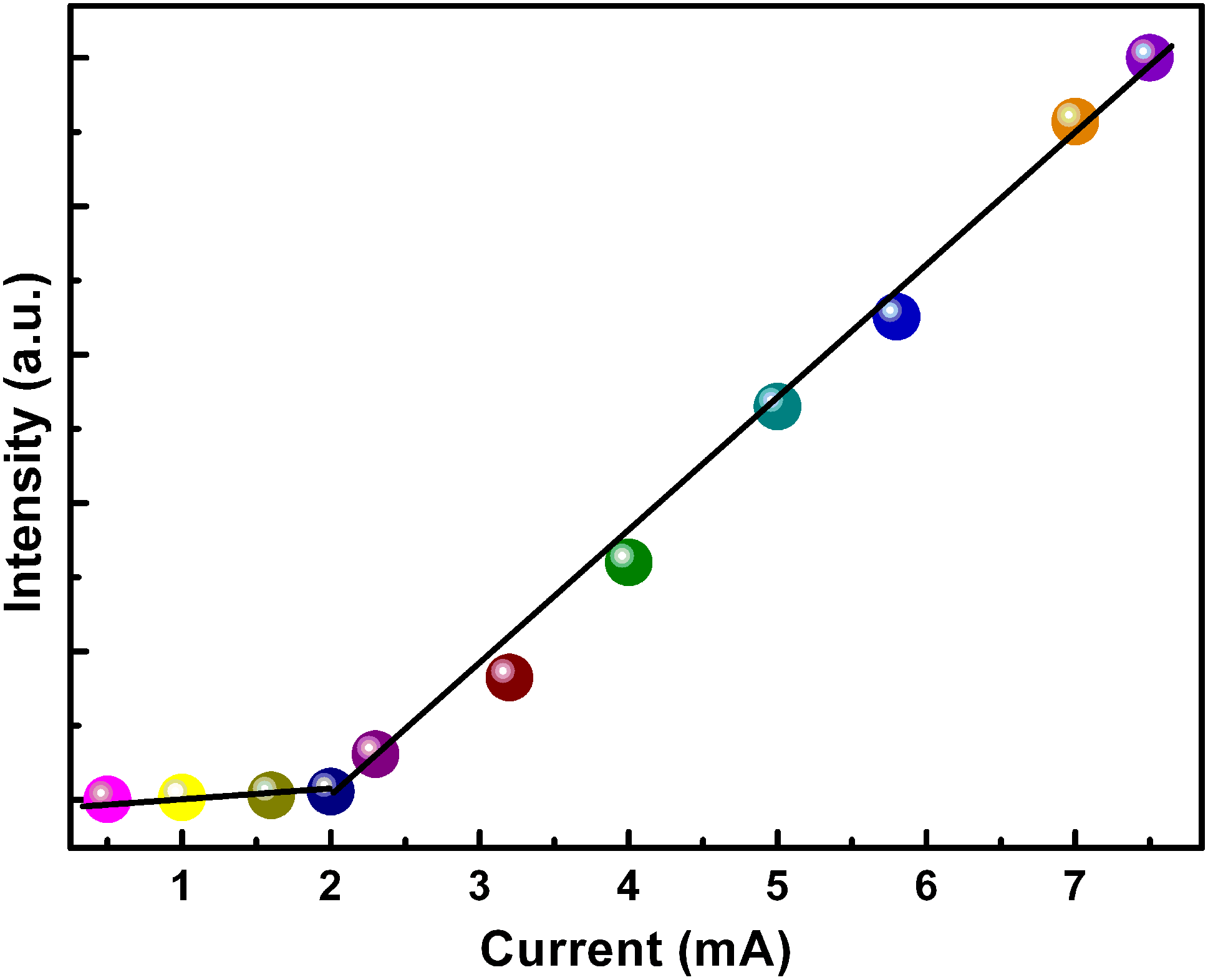


**Figure S4.** Dependence of the integrated EL intensity of the laser diode on injection current at RT.

As shown in Fig. S4, the dependence of the integrated EL intensity *versus* injection current is presented, from which a threshold current of ~2.1 mA is derived, corresponding to an ultralow current density of 0.27 A/cm^2^.

**Introduction of ZnO NNWs structures**

We have done parametric studies on the controllable growth of high-quality ZnO NNWs by catalyst-free MOCVD system. By adjusting the growth parameters in specified windows, ZnO NNWs with different microcavity size and wall thickness can be selectively obtained (*CrystEngComm*, **16**, 455-463, 2014).

In order to assess the suitability of produced ZnO NNWs (with a typical wall thickness of ~40 nm) to practical light-emitting diodes (LEDs) application, hetero-epitaxial n-ZnO NNWs/p-GaN structure was constructed (*Appl. Phys. Lett.*, **103**, 021109, 2013). A highly-efficient UV-blue emission can be obtained with a low driving current, and the heterojunction diode can be operated continuously for 8.5 h with a decay of only 8.08% under a continuous current of 12 mA. This work indicates that vertically aligned ZnO NNWs can be employed as an effective emitting layer in LEDs. Maybe it is because the absence of carrier confinement configuration (the energy barriers at ZnO/GaN interface for electrons and holes are relatively small and comparably equal)^1^, no lasing action was observed in such heterostructures.

1. Lupan, O., Pauporté, T. & Viana, B. Low-voltage UV-electroluminescence from ZnO-nanowire array/p-GaN light-emitting diodes. *Adv. Mater.* **22**, 3298-3302 (2010).
